# (7-Isopropyl-1,4a-dimethyl-1,2,3,4,4a,-9,10,10a-octa­hydro­phenanthren-1-yl)­methanaminium 4-toluene­sulfonate

**DOI:** 10.1107/S1600536808010210

**Published:** 2008-04-18

**Authors:** Yong Li, Tao Zeng, Dao-zhan Huang

**Affiliations:** a College of Chemical Engineering, Nanjing Forestry University, Nanjing 210037, People’s Republic of China

## Abstract

In the title compound, C_20_H_32_N^+^·C_7_H_7_O_3_S^−^, the configurations of the two chiral centers observed in the protonated cation are consistent with previous reports. In the crystal structure, weak inter­molecular N—H⋯O hydrogen bonds link ions into chains which develop along the *a* axis. The isopropyl group and four CH groups of the attached benzene ring are disordered approximately equally over two positions.

## Related literature

For related literature, see: Gottstein & Cheney (1965 ▶); Rao *et al.* (2006 ▶); Tao (1993 ▶).
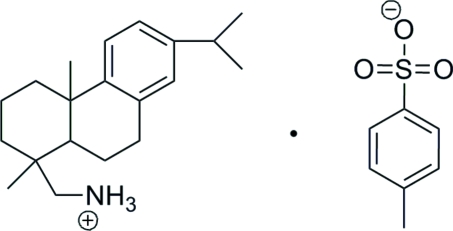


## Experimental

### 

#### Crystal data

C_20_H_32_N^+^·C_7_H_7_O_3_S^−^
*M* *_r_* = 457.65
Orthorhombic, 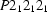

*a* = 5.9954 (2) Å
*b* = 11.7039 (5) Å
*c* = 37.0381 (13) Å
*V* = 2598.95 (17) Å^3^
*Z* = 4
Mo *K*α radiation
μ = 0.15 mm^−1^
*T* = 296 (2) K
0.40 × 0.30 × 0.30 mm

#### Data collection

Bruker SMART CCD area-detector diffractometer
Absorption correction: multi-scan (*SADABS*; Bruker, 2000 ▶) *T*_min_ = 0.942, *T*_max_ = 0.956
22386 measured reflections
5925 independent reflections
4346 reflections with *I* > 2σ(*I*)
*R*_int_ = 0.029

#### Refinement

*R*[*F* ^2^ > 2σ(*F* ^2^)] = 0.045
*wR*(*F* ^2^) = 0.139
*S* = 1.05
5925 reflections
360 parameters
204 restraints
H-atom parameters constrained
Δρ_max_ = 0.22 e Å^−3^
Δρ_min_ = −0.35 e Å^−3^
Absolute structure: Flack (1983 ▶), 2489 Friedel pairs
Flack parameter: 0.03 (8)

### 

Data collection: *SMART* (Bruker, 2002 ▶); cell refinement: *SAINT-Plus* (Bruker, 2003 ▶); data reduction: *SAINT-Plus*; program(s) used to solve structure: *SHELXTL* (Sheldrick, 2008 ▶); program(s) used to refine structure: *SHELXL97* (Sheldrick, 2008 ▶); molecular graphics: *ORTEPIII* (Burnett & Johnson, 1996 ▶), *ORTEP3* (Farrugia, 1997 ▶) and *XP* in *SHELXTL*; software used to prepare material for publication: *SHELXTL*.

## Supplementary Material

Crystal structure: contains datablocks I, global. DOI: 10.1107/S1600536808010210/dn2336sup1.cif

Structure factors: contains datablocks I. DOI: 10.1107/S1600536808010210/dn2336Isup2.hkl

Additional supplementary materials:  crystallographic information; 3D view; checkCIF report

## Figures and Tables

**Table 1:** Hydrogen-bond geometry (Å, °)

*D*—H⋯*A*	*D*—H	H⋯*A*	*D*⋯*A*	*D*—H⋯*A*
N1—H1*C*⋯O1	0.89	2.06	2.835 (3)	145
N1—H1*D*⋯O3^i^	0.89	1.84	2.722 (3)	173
N1—H1*E*⋯O1^ii^	0.89	1.95	2.772 (3)	152

Symmetry codes: (i) 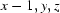; (ii) 
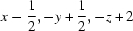
.

**Fig. 1.**
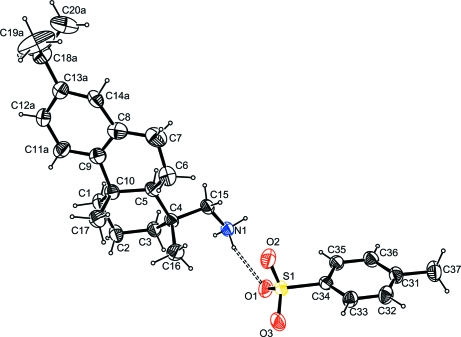
A view of the title compound with the atom-labelling scheme. Thermal ellipsoids are drawn at the 30% probability level. H atoms are represented as small spheres of arbitrary radii. Only one aprt of the disordered moiety is represented for clarity. H bond is shown as dashed line.

## supplementary crystallographic information

### Comment

Dehydroabietylamine, a unique synthetic primary amine having a three fused rings structure, is obtained as part of a mixture of amines prepared by the hydrogenation of rosin acid nitrile.

The cation and anion are linked by N-H···O hydrogen bond (Table 1, Fig.1). The *R* and *S* absolute configuration observed at C4 and C10 has been determined by the refinement of the Flack parameter (Flack, 1983). These absolute configurations agree with previous reports(Rao *et al.*, 2006, Tao, 1993).

Further N—H···O hydrogen bonds between the amine group and the O atoms of the SO_3_ group, link molecules into chains developing along the *a* axis (Table 1).

### Experimental

The title compound was synthesized according to the literature method(Gottstein & Cheney, 1965, Tao, 1993).

### Refinement

All H atoms attached to C atoms and N atom were fixed geometrically and treated as riding with C—H = 0.98 Å (methine), 0.97 Å(methylene), 0.96Å(methyl) or 0.93Å (aromatic) and N—H = 0.89Å with U_iso_(H) = 1.2U_eq_(C) or U_iso_(H) = 1.5U_eq_(C_methyl_, N).

The isopropyl group and part of the ring to which it is attached is  disordered over two positions as indicated by very elongated thermal ellipsoids. This disorder was treated using the PART and SADI instruction available in SHELXL97 (Sheldrick, 2008). The ratio between the two disordered fragments has been initialy obtained by refining the occupancy factor using the FVAR instruction with an overall isotropic thermal parameter. The ratio of the occupancy factor was found to be 0.53/0.47. Once correctly defined, the occupancy factors were fixed and a full refinement using restraints on C-C distances (SADI) and U_IJ_ (SIMU, DELU) was carried out.

### Crystal data

**Table:**

C_20_H_32_N^+^·C_7_H_7_O_3_S^–^	*F*_000_ = 992
*M**_r_* = 457.65	*D*_x_ = 1.170 Mg m^−^^3^
Orthorhombic, *P*2_1_2_1_2_1_	Mo *K*α radiation λ = 0.71073 Å
Hall symbol: P 2ac 2ab	Cell parameters from 5550 reflections
*a* = 5.9954 (2) Å	θ = 2.4–21.4º
*b* = 11.7039 (5) Å	µ = 0.15 mm^−^^1^
*c* = 37.0381 (13) Å	*T* = 296 (2) K
*V* = 2598.95 (17) Å^3^	Box, colourless
*Z* = 4	0.40 × 0.30 × 0.30 mm

### Data collection

**Table:**

Bruker SMART CCD area-detector diffractometer	5925 independent reflections
Radiation source: fine-focus sealed tube	4346 reflections with *I* > 2σ(*I*)
Monochromator: graphite	*R*_int_ = 0.029
*T* = 296(2) K	θ_max_ = 27.5º
φ and ω scans	θ_min_ = 1.1º
Absorption correction: multi-scan(SADABS; Bruker, 2000)	*h* = −7→7
*T*_min_ = 0.942, *T*_max_ = 0.956	*k* = −15→10
22386 measured reflections	*l* = −45→48

### Refinement

**Table:**

Refinement on *F*^2^	Hydrogen site location: inferred from neighbouring sites
Least-squares matrix: full	H-atom parameters constrained
*R*[*F*^2^ > 2σ(*F*^2^)] = 0.045	*w* = 1/[σ^2^(*F*_o_^2^) + (0.0817*P*)^2^] where *P* = (*F*_o_^2^ + 2*F*_c_^2^)/3
*wR*(*F*^2^) = 0.139	(Δ/σ)_max_ = 0.001
*S* = 1.05	Δρ_max_ = 0.22 e Å^−^^3^
5925 reflections	Δρ_min_ = −0.35 e Å^−^^3^
360 parameters	Extinction correction: none
204 restraints	Absolute structure: Flack (1983), 2489 Friedel pairs
Primary atom site location: structure-invariant direct methods	Flack parameter: 0.03 (8)
Secondary atom site location: difference Fourier map	

### Special details

**Table:**

Geometry. All esds (except the esd in the dihedral angle between two l.s. planes) are estimated using the full covariance matrix. The cell esds are taken into account individually in the estimation of esds in distances, angles and torsion angles; correlations between esds in cell parameters are only used when they are defined by crystal symmetry. An approximate (isotropic) treatment of cell esds is used for estimating esds involving l.s. planes.
Refinement. Refinement of *F*^2^ against ALL reflections. The weighted *R*-factor *wR* and goodness of fit *S* are based on *F*^2^, conventional *R*-factors *R* are based on *F*, with *F* set to zero for negative *F*^2^. The threshold expression of *F*^2^ > σ(*F*^2^) is used only for calculating *R*-factors(gt) *etc*. and is not relevant to the choice of reflections for refinement. *R*-factors based on *F*^2^ are statistically about twice as large as those based on *F*, and *R*- factors based on ALL data will be even larger.

### Fractional atomic coordinates and isotropic or equivalent isotropic displacement parameters (Å^2^)

**Table:**

	*x*	*y*	*z*	*U*_iso_*/*U*_eq_	Occ. (<1)
C1	0.4813 (5)	0.45313 (18)	0.84444 (6)	0.0648 (6)	
H1A	0.5164	0.5149	0.8279	0.078*	
H1B	0.3210	0.4529	0.8481	0.078*	
C2	0.5952 (5)	0.4754 (2)	0.88024 (6)	0.0714 (7)	
H2A	0.5444	0.5479	0.8899	0.086*	
H2B	0.7551	0.4804	0.8766	0.086*	
C3	0.5442 (4)	0.38020 (18)	0.90724 (6)	0.0596 (6)	
H3A	0.6241	0.3956	0.9295	0.072*	
H3B	0.3860	0.3813	0.9127	0.072*	
C4	0.6073 (3)	0.26118 (18)	0.89395 (6)	0.0513 (5)	
C5	0.5098 (3)	0.24264 (17)	0.85538 (5)	0.0512 (5)	
H5	0.3478	0.2425	0.8589	0.061*	
C6	0.5598 (5)	0.1269 (2)	0.83848 (7)	0.0723 (7)	
H6A	0.7071	0.1275	0.8277	0.087*	
H6B	0.5562	0.0678	0.8568	0.087*	
C7	0.3844 (6)	0.1028 (3)	0.80977 (8)	0.0937 (10)	
H7A	0.4337	0.0386	0.7952	0.112*	
H7B	0.2467	0.0806	0.8216	0.112*	
C8	0.3379 (5)	0.2025 (3)	0.78531 (7)	0.0763 (8)	
C9	0.4075 (4)	0.3128 (2)	0.79388 (6)	0.0643 (6)	
C10	0.5512 (4)	0.3390 (2)	0.82710 (6)	0.0557 (5)	
C11A	0.354 (6)	0.4122 (15)	0.7717 (7)	0.073 (4)	0.47
H11A	0.4093	0.4832	0.7787	0.087*	0.47
C12A	0.2225 (19)	0.4056 (6)	0.7397 (3)	0.065 (2)	0.47
H12A	0.1884	0.4708	0.7265	0.078*	0.47
C13A	0.1461 (16)	0.2979 (8)	0.7289 (2)	0.061 (2)	0.47
C14A	0.210 (2)	0.2074 (7)	0.7520 (3)	0.066 (2)	0.47
H14A	0.1599	0.1364	0.7442	0.079*	0.47
C18A	0.0100 (12)	0.2855 (7)	0.69557 (16)	0.0750 (17)	0.47
H18A	−0.0248	0.3610	0.6856	0.090*	0.47
C19A	0.108 (2)	0.2104 (16)	0.6668 (4)	0.164 (8)	0.47
H19A	0.1331	0.1354	0.6764	0.246*	0.47
H19B	0.2467	0.2422	0.6587	0.246*	0.47
H19C	0.0062	0.2056	0.6468	0.246*	0.47
C20A	−0.199 (3)	0.2203 (19)	0.7017 (7)	0.178 (10)	0.47
H20A	−0.1629	0.1465	0.7114	0.268*	0.47
H20B	−0.2766	0.2114	0.6793	0.268*	0.47
H20C	−0.2912	0.2610	0.7185	0.268*	0.47
C11B	0.344 (6)	0.3896 (14)	0.7680 (6)	0.076 (4)	0.53
H11B	0.3803	0.4664	0.7707	0.091*	0.53
C12B	0.2278 (18)	0.3544 (7)	0.7386 (3)	0.069 (2)	0.53
H12B	0.1855	0.4096	0.7218	0.083*	0.53
C13B	0.1685 (14)	0.2434 (7)	0.7317 (3)	0.068 (2)	0.53
C14B	0.223 (2)	0.1613 (6)	0.7559 (2)	0.068 (2)	0.53
H14B	0.1867	0.0847	0.7529	0.082*	0.53
C18B	0.0331 (11)	0.2128 (8)	0.69788 (16)	0.0893 (19)	0.53
H18B	0.0283	0.1295	0.6954	0.107*	0.53
C19B	0.162 (2)	0.2641 (14)	0.6647 (3)	0.136 (6)	0.53
H19D	0.1625	0.3460	0.6664	0.205*	0.53
H19E	0.0897	0.2412	0.6428	0.205*	0.53
H19F	0.3129	0.2365	0.6648	0.205*	0.53
C20B	−0.2109 (16)	0.2584 (13)	0.7026 (4)	0.094 (3)	0.53
H20D	−0.2664	0.2366	0.7259	0.141*	0.53
H20E	−0.3045	0.2264	0.6842	0.141*	0.53
H20F	−0.2113	0.3402	0.7006	0.141*	0.53
C15	0.4892 (4)	0.17224 (18)	0.91797 (6)	0.0576 (5)	
H15A	0.3300	0.1765	0.9134	0.069*	
H15B	0.5387	0.0967	0.9108	0.069*	
C16	0.8591 (4)	0.2415 (3)	0.89661 (8)	0.0758 (7)	
H16A	0.9361	0.3014	0.8840	0.114*	
H16B	0.8960	0.1692	0.8860	0.114*	
H16C	0.9031	0.2417	0.9215	0.114*	
C17	0.7940 (4)	0.3494 (3)	0.81380 (7)	0.0770 (8)	
H17A	0.8009	0.4033	0.7943	0.115*	
H17B	0.8453	0.2762	0.8056	0.115*	
H17C	0.8870	0.3752	0.8333	0.115*	
C31	0.8536 (4)	−0.25930 (19)	1.06820 (6)	0.0626 (6)	
C32	1.0535 (4)	−0.25420 (19)	1.04994 (6)	0.0678 (7)	
H32	1.1605	−0.3103	1.0540	0.081*	
C33	1.0981 (4)	−0.16706 (19)	1.02573 (6)	0.0598 (6)	
H33	1.2343	−0.1647	1.0137	0.072*	
C34	0.9414 (3)	−0.08452 (17)	1.01948 (6)	0.0506 (5)	
C35	0.7373 (4)	−0.08909 (19)	1.03689 (6)	0.0607 (6)	
H35	0.6292	−0.0339	1.0324	0.073*	
C36	0.6962 (4)	−0.1768 (2)	1.06106 (7)	0.0667 (6)	
H36	0.5590	−0.1801	1.0727	0.080*	
C37	0.8088 (7)	−0.3537 (2)	1.09495 (8)	0.0913 (10)	
H37A	0.7348	−0.4158	1.0830	0.137*	
H37B	0.7157	−0.3253	1.1140	0.137*	
H37C	0.9475	−0.3801	1.1049	0.137*	
N1	0.5270 (3)	0.18534 (15)	0.95701 (5)	0.0565 (4)	
H1C	0.6678	0.1678	0.9622	0.085*	
H1D	0.4359	0.1389	0.9690	0.085*	
H1E	0.5001	0.2573	0.9634	0.085*	
O1	0.8894 (4)	0.12792 (13)	1.00269 (5)	0.0828 (6)	
O2	0.9025 (4)	−0.00303 (15)	0.95411 (4)	0.0818 (5)	
O3	1.2366 (3)	0.0371 (2)	0.98855 (8)	0.1196 (10)	
S1	0.99852 (11)	0.02625 (5)	0.988456 (17)	0.06402 (19)	

### Atomic displacement parameters (Å^2^)

**Table:**

	*U*^11^	*U*^22^	*U*^33^	*U*^12^	*U*^13^	*U*^23^
C1	0.0798 (17)	0.0532 (12)	0.0612 (13)	−0.0036 (13)	0.0047 (13)	0.0070 (10)
C2	0.0970 (19)	0.0557 (13)	0.0615 (14)	−0.0134 (14)	0.0079 (13)	0.0041 (11)
C3	0.0707 (15)	0.0544 (11)	0.0537 (12)	−0.0089 (11)	0.0018 (11)	0.0018 (9)
C4	0.0412 (10)	0.0523 (11)	0.0604 (12)	−0.0054 (10)	0.0072 (9)	0.0037 (10)
C5	0.0432 (10)	0.0515 (10)	0.0589 (12)	−0.0003 (10)	0.0120 (10)	−0.0024 (9)
C6	0.086 (2)	0.0623 (14)	0.0682 (15)	0.0065 (13)	0.0188 (14)	−0.0090 (11)
C7	0.111 (2)	0.0824 (19)	0.088 (2)	−0.0165 (19)	0.0141 (19)	−0.0295 (17)
C8	0.0679 (16)	0.100 (2)	0.0611 (16)	−0.0084 (16)	0.0129 (12)	−0.0205 (15)
C9	0.0513 (12)	0.0902 (18)	0.0513 (13)	−0.0044 (13)	0.0133 (11)	−0.0015 (12)
C10	0.0511 (13)	0.0661 (13)	0.0499 (12)	−0.0036 (10)	0.0088 (9)	0.0011 (10)
C11A	0.064 (7)	0.101 (7)	0.053 (6)	0.003 (7)	−0.002 (4)	0.007 (6)
C12A	0.071 (4)	0.061 (4)	0.062 (4)	0.009 (5)	0.006 (3)	0.011 (4)
C13A	0.057 (4)	0.071 (6)	0.055 (4)	0.009 (4)	0.011 (3)	0.000 (4)
C14A	0.077 (5)	0.049 (5)	0.071 (5)	−0.013 (5)	0.013 (4)	−0.004 (5)
C18A	0.076 (4)	0.082 (4)	0.068 (4)	0.014 (4)	−0.008 (3)	−0.006 (3)
C19A	0.113 (10)	0.257 (18)	0.121 (10)	0.111 (12)	−0.046 (8)	−0.101 (10)
C20A	0.162 (18)	0.182 (19)	0.191 (15)	−0.089 (14)	−0.055 (12)	−0.012 (13)
C11B	0.069 (6)	0.098 (7)	0.060 (6)	−0.014 (6)	0.008 (5)	−0.001 (5)
C12B	0.079 (5)	0.069 (5)	0.058 (4)	0.021 (5)	−0.001 (3)	0.002 (5)
C13B	0.061 (3)	0.077 (6)	0.067 (4)	0.013 (4)	0.003 (3)	−0.010 (4)
C14B	0.083 (4)	0.058 (4)	0.063 (4)	−0.014 (5)	0.013 (3)	−0.011 (4)
C18B	0.091 (5)	0.100 (5)	0.077 (4)	0.023 (5)	−0.012 (3)	−0.015 (4)
C19B	0.109 (7)	0.253 (15)	0.047 (4)	−0.027 (8)	−0.010 (4)	−0.024 (6)
C20B	0.068 (5)	0.124 (8)	0.091 (6)	0.002 (5)	−0.020 (4)	−0.005 (5)
C15	0.0590 (13)	0.0555 (11)	0.0583 (12)	−0.0110 (11)	0.0052 (11)	0.0063 (9)
C16	0.0469 (13)	0.0997 (19)	0.0810 (17)	0.0028 (14)	0.0065 (11)	0.0155 (16)
C17	0.0577 (15)	0.107 (2)	0.0661 (15)	−0.0105 (14)	0.0169 (12)	0.0118 (15)
C31	0.0839 (17)	0.0460 (11)	0.0578 (13)	−0.0055 (12)	−0.0035 (11)	0.0019 (10)
C32	0.0838 (18)	0.0469 (11)	0.0728 (15)	0.0147 (12)	−0.0042 (13)	0.0028 (11)
C33	0.0549 (13)	0.0567 (13)	0.0679 (14)	0.0115 (10)	0.0036 (11)	0.0048 (10)
C34	0.0461 (11)	0.0456 (10)	0.0601 (12)	−0.0033 (9)	−0.0021 (9)	0.0019 (9)
C35	0.0482 (12)	0.0524 (12)	0.0816 (16)	0.0031 (10)	−0.0028 (12)	0.0089 (11)
C36	0.0612 (14)	0.0653 (14)	0.0735 (15)	−0.0078 (12)	0.0075 (12)	0.0065 (12)
C37	0.137 (3)	0.0662 (16)	0.0708 (17)	−0.0128 (17)	−0.0046 (18)	0.0159 (13)
N1	0.0503 (10)	0.0550 (9)	0.0641 (11)	−0.0047 (8)	0.0032 (9)	0.0131 (8)
O1	0.1152 (16)	0.0473 (9)	0.0859 (12)	−0.0018 (10)	−0.0308 (11)	0.0102 (8)
O2	0.1068 (14)	0.0746 (11)	0.0641 (10)	0.0038 (10)	−0.0039 (10)	0.0154 (9)
O3	0.0561 (11)	0.1249 (19)	0.178 (2)	−0.0252 (12)	−0.0091 (13)	0.0906 (18)
S1	0.0572 (3)	0.0545 (3)	0.0804 (4)	−0.0071 (3)	−0.0065 (3)	0.0208 (3)

### Geometric parameters (Å, °)

**Table:**

C1—C2	1.514 (3)	C11B—H11B	0.9300
C1—C10	1.540 (3)	C12B—C13B	1.371 (9)
C1—H1A	0.9700	C12B—H12B	0.9300
C1—H1B	0.9700	C13B—C14B	1.353 (8)
C2—C3	1.528 (3)	C13B—C18B	1.535 (10)
C2—H2A	0.9700	C14B—H14B	0.9300
C2—H2B	0.9700	C18B—C20B	1.566 (11)
C3—C4	1.525 (3)	C18B—C19B	1.571 (12)
C3—H3A	0.9700	C18B—H18B	0.9800
C3—H3B	0.9700	C19B—H19D	0.9600
C4—C16	1.530 (3)	C19B—H19E	0.9600
C4—C15	1.542 (3)	C19B—H19F	0.9600
C4—C5	1.559 (3)	C20B—H20D	0.9600
C5—C6	1.522 (3)	C20B—H20E	0.9600
C5—C10	1.559 (3)	C20B—H20F	0.9600
C5—H5	0.9800	C15—N1	1.472 (3)
C6—C7	1.522 (4)	C15—H15A	0.9700
C6—H6A	0.9700	C15—H15B	0.9700
C6—H6B	0.9700	C16—H16A	0.9600
C7—C8	1.504 (4)	C16—H16B	0.9600
C7—H7A	0.9700	C16—H16C	0.9600
C7—H7B	0.9700	C17—H17A	0.9600
C8—C14B	1.375 (9)	C17—H17B	0.9600
C8—C9	1.393 (4)	C17—H17C	0.9600
C8—C14A	1.455 (10)	C31—C36	1.376 (3)
C9—C11B	1.368 (11)	C31—C32	1.377 (4)
C9—C11A	1.460 (12)	C31—C37	1.508 (3)
C9—C10	1.533 (3)	C32—C33	1.384 (3)
C10—C17	1.542 (3)	C32—H32	0.9300
C11A—C12A	1.425 (11)	C33—C34	1.367 (3)
C11A—H11A	0.9300	C33—H33	0.9300
C12A—C13A	1.400 (9)	C34—C35	1.384 (3)
C12A—H12A	0.9300	C34—S1	1.766 (2)
C13A—C14A	1.412 (9)	C35—C36	1.384 (3)
C13A—C18A	1.488 (11)	C35—H35	0.9300
C14A—H14A	0.9300	C36—H36	0.9300
C18A—C20A	1.482 (16)	C37—H37A	0.9600
C18A—C19A	1.501 (12)	C37—H37B	0.9600
C18A—H18A	0.9800	C37—H37C	0.9600
C19A—H19A	0.9600	N1—H1C	0.8900
C19A—H19B	0.9600	N1—H1D	0.8900
C19A—H19C	0.9600	N1—H1E	0.8900
C20A—H20A	0.9600	O1—S1	1.457 (2)
C20A—H20B	0.9600	O2—S1	1.4377 (19)
C20A—H20C	0.9600	O3—S1	1.433 (2)
C11B—C12B	1.359 (11)		
			
C2—C1—C10	113.1 (2)	C11B—C12B—C13B	124.7 (9)
C2—C1—H1A	109.0	C11B—C12B—H12B	117.7
C10—C1—H1A	109.0	C13B—C12B—H12B	117.7
C2—C1—H1B	109.0	C14B—C13B—C12B	119.2 (7)
C10—C1—H1B	109.0	C14B—C13B—C18B	120.2 (7)
H1A—C1—H1B	107.8	C12B—C13B—C18B	120.6 (8)
C1—C2—C3	110.9 (2)	C13B—C14B—C8	113.4 (6)
C1—C2—H2A	109.5	C13B—C14B—H14B	123.3
C3—C2—H2A	109.5	C8—C14B—H14B	123.3
C1—C2—H2B	109.5	C13B—C18B—C20B	108.9 (7)
C3—C2—H2B	109.5	C13B—C18B—C19B	106.8 (8)
H2A—C2—H2B	108.0	C20B—C18B—C19B	114.6 (9)
C4—C3—C2	113.92 (18)	C13B—C18B—H18B	108.8
C4—C3—H3A	108.8	C20B—C18B—H18B	108.8
C2—C3—H3A	108.8	C19B—C18B—H18B	108.8
C4—C3—H3B	108.8	C18B—C19B—H19D	109.5
C2—C3—H3B	108.8	C18B—C19B—H19E	109.5
H3A—C3—H3B	107.7	H19D—C19B—H19E	109.5
C3—C4—C16	111.2 (2)	C18B—C19B—H19F	109.5
C3—C4—C15	108.46 (17)	H19D—C19B—H19F	109.5
C16—C4—C15	108.3 (2)	H19E—C19B—H19F	109.5
C3—C4—C5	109.27 (17)	C18B—C20B—H20D	109.5
C16—C4—C5	114.08 (19)	C18B—C20B—H20E	109.5
C15—C4—C5	105.23 (17)	H20D—C20B—H20E	109.5
C6—C5—C4	115.28 (18)	C18B—C20B—H20F	109.5
C6—C5—C10	109.66 (17)	H20D—C20B—H20F	109.5
C4—C5—C10	117.08 (17)	H20E—C20B—H20F	109.5
C6—C5—H5	104.4	N1—C15—C4	115.21 (18)
C4—C5—H5	104.4	N1—C15—H15A	108.5
C10—C5—H5	104.4	C4—C15—H15A	108.5
C7—C6—C5	108.5 (2)	N1—C15—H15B	108.5
C7—C6—H6A	110.0	C4—C15—H15B	108.5
C5—C6—H6A	110.0	H15A—C15—H15B	107.5
C7—C6—H6B	110.0	C4—C16—H16A	109.5
C5—C6—H6B	110.0	C4—C16—H16B	109.5
H6A—C6—H6B	108.4	H16A—C16—H16B	109.5
C8—C7—C6	113.9 (2)	C4—C16—H16C	109.5
C8—C7—H7A	108.8	H16A—C16—H16C	109.5
C6—C7—H7A	108.8	H16B—C16—H16C	109.5
C8—C7—H7B	108.8	C10—C17—H17A	109.5
C6—C7—H7B	108.8	C10—C17—H17B	109.5
H7A—C7—H7B	107.7	H17A—C17—H17B	109.5
C14B—C8—C9	130.9 (4)	C10—C17—H17C	109.5
C14B—C8—C14A	22.8 (3)	H17A—C17—H17C	109.5
C9—C8—C14A	108.3 (4)	H17B—C17—H17C	109.5
C14B—C8—C7	107.4 (4)	C36—C31—C32	118.2 (2)
C9—C8—C7	121.8 (2)	C36—C31—C37	121.2 (3)
C14A—C8—C7	129.8 (4)	C32—C31—C37	120.6 (2)
C11B—C9—C8	111.5 (7)	C31—C32—C33	121.2 (2)
C11B—C9—C11A	11.7 (14)	C31—C32—H32	119.4
C8—C9—C11A	123.0 (7)	C33—C32—H32	119.4
C11B—C9—C10	125.9 (7)	C34—C33—C32	119.9 (2)
C8—C9—C10	122.4 (2)	C34—C33—H33	120.1
C11A—C9—C10	114.5 (7)	C32—C33—H33	120.1
C9—C10—C1	110.8 (2)	C33—C34—C35	120.1 (2)
C9—C10—C17	106.86 (19)	C33—C34—S1	119.73 (17)
C1—C10—C17	108.8 (2)	C35—C34—S1	120.19 (17)
C9—C10—C5	107.74 (19)	C34—C35—C36	119.2 (2)
C1—C10—C5	107.70 (16)	C34—C35—H35	120.4
C17—C10—C5	115.0 (2)	C36—C35—H35	120.4
C12A—C11A—C9	123.0 (13)	C31—C36—C35	121.5 (2)
C12A—C11A—H11A	118.5	C31—C36—H36	119.3
C9—C11A—H11A	118.5	C35—C36—H36	119.3
C13A—C12A—C11A	117.8 (10)	C31—C37—H37A	109.5
C13A—C12A—H12A	121.1	C31—C37—H37B	109.5
C11A—C12A—H12A	121.1	H37A—C37—H37B	109.5
C12A—C13A—C14A	114.5 (7)	C31—C37—H37C	109.5
C12A—C13A—C18A	120.3 (7)	H37A—C37—H37C	109.5
C14A—C13A—C18A	125.3 (8)	H37B—C37—H37C	109.5
C13A—C14A—C8	133.3 (7)	C15—N1—H1C	109.5
C13A—C14A—H14A	113.4	C15—N1—H1D	109.5
C8—C14A—H14A	113.4	H1C—N1—H1D	109.5
C20A—C18A—C13A	112.6 (11)	C15—N1—H1E	109.5
C20A—C18A—C19A	97.9 (13)	H1C—N1—H1E	109.5
C13A—C18A—C19A	115.6 (8)	H1D—N1—H1E	109.5
C20A—C18A—H18A	110.0	O3—S1—O2	114.95 (16)
C13A—C18A—H18A	110.0	O3—S1—O1	111.98 (17)
C19A—C18A—H18A	110.0	O2—S1—O1	109.59 (11)
C12B—C11B—C9	120.4 (12)	O3—S1—C34	104.85 (11)
C12B—C11B—H11B	119.8	O2—S1—C34	108.85 (10)
C9—C11B—H11B	119.8	O1—S1—C34	106.09 (11)

### Hydrogen-bond geometry (Å, °)

**Table:**

*D*—H···*A*	*D*—H	H···*A*	*D*···*A*	*D*—H···*A*
N1—H1C···O1	0.89	2.06	2.835 (3)	145
N1—H1D···O3^i^	0.89	1.84	2.722 (3)	173
N1—H1E···O1^ii^	0.89	1.95	2.772 (3)	152

Symmetry codes: (i) *x*−1, *y*, *z*; (ii) *x*−1/2, −*y*+1/2, −*z*+2.
